# Neuroprotective Mechanisms of Puerarin in Central Nervous System Diseases: Update

**DOI:** 10.14336/AD.2021.1205

**Published:** 2022-07-11

**Authors:** Chao-Chao Yu, Yan-Jun Du, Jin Li, Yi Li, Li Wang, Li-Hong Kong, Ying-Wen Zhang

**Affiliations:** ^1^Department of Integrated Chinese and Western Medicine, Zhongnan Hospital of Wuhan University, Wuhan University, Wuhan, Hubei, China.; ^2^College of Acupuncture and Orthopedics, Hubei University of Chinese Medicine, Wuhan, Hubei, China.; ^3^Fourth Clinical Medical College, Guangzhou University of Chinese Medicine, Shenzhen, Guangdong, China.; ^4^Department of Oncology, Zhongnan Hospital of Wuhan University, Wuhan University, Wuhan, Hubei, China.

**Keywords:** puerarin, Alzheimer’s disease, Parkinson’s disease, cerebral ischemia, depression, spinal cord injury

## Abstract

Due to global population aging and modern lifestyle changes, the incidence of central nervous system (CNS) disorders, such as neurodegenerative diseases, neuropsychiatric disorders, and cerebrovascular diseases, is increasing and has become a major public health challenge. Current medications commonly used in the clinic are far from satisfactory and may cause serious side effects. Therefore, the identification of novel drugs for the effective management of CNS diseases is very urgent. Puerarin, a highly bioactive ingredient isolated from *Pueraria lobata*, is known to possess a broad spectrum of pharmacological properties including anti-diabetic, anti-inflammatory, anti-antioxidant, neuroprotective, and cardioprotective features. However, its clinical application is limited due to its poor water solubility. Since puerarin has demonstrated a wide range of neuroprotective functions in various CNS diseases, such as Alzheimer’s disease, Parkinson’s disease, cerebral ischemia, depression, and spinal cord injury, it has been attracting increasingly intense attention worldwide. In this review, we intend to extensively summarize the research progress on neuroprotective mechanisms of puerarin in recent years and discuss the future directions of its application in CNS disease treatment.

## 1. Introduction

Puerarin is the main active constituent isolated from the root of the *Pueraria lobata* (Willd.) *Ohwi* (Fabaceae), which is native to Southeast Asia and widely known as *Gegen* in traditional Chinese medicine. As one of the earliest known Chinese medicinal herbs in China, *Gegen* is frequently used to treat a wide range of conditions including fever, pain, diabetes, gastrointestinal diseases, cerebrovascular disorders, and cardiac dysfunctions [1]. Among various compounds isolated from *Gegen*, puerarin is considered to be the major active ingredient responsible for exerting its pharmacological effect [2]. In recent decades, numerous studies have demonstrated that puerarin possesses significant therapeutic effects for various kinds of central nervous system (CNS) diseases, such as Alzheimer’s disease (AD), Parkinson’s disease (PD), cerebral ischemia, depression, and spinal cord injury. In the current review, to provide insights into the discovery and development of novel neuroprotective agents, we discuss the structural features of puerarin and comprehensively summarize the current knowledge on its pharmacological mechanisms of action against CNS diseases.

## 2. Chemico-Structural Characteristics of Puerarin

**Figure 1.**
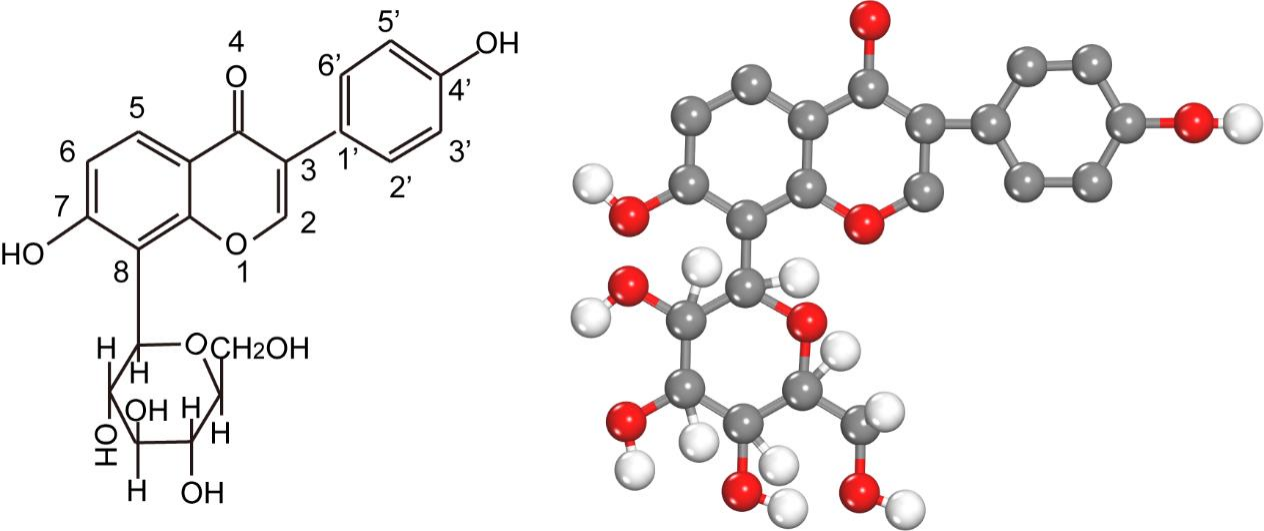
**The chemical structure of puerarin**. In the ball and stick model, grey, red, and white balls represent carbon, oxygen, and hydrogen atoms, respectively.

Puerarin is chemically known as 7,4’-dihydroxy-8-C-glucosylisoflavone (Fig. 1), with a molecular formula of C_21_H_20_O_9_[2]. Puerarin has a glucopyranose attached to the 8-position and a hydroxyl group to each of the 7,4'-positions, which are the chemical structures related to its pharmacological actions. However, the 7-position hydroxyl group is less active than one at the 4-position due to the site blocking effect of the 8-position glucosyl group. The glucosyl group is considered to be the structural basis for water solubility of puerarin. Alternatively, the two hydroxyl groups and the carbonyl group at the 4-position may constitute sites for interaction with the co-solvent. This chemico-structural property of puerarin contributes to its poor water-solubility and liposolubility, thus leading to poor oral absorption and low bioavailability, which eventually restricts its wider clinical application. Besides, the pH also has an impact on the solubility of puerarin, which is 0.46 mg/mL in an aqueous solution and can reach a maximum of 7.56 mg/mL at a pH of 7.4 in phosphate buffers [3,4]. To improve the solubility of puerarin, co-solvents such as ethylene glycol, polyvinylpyrrolidone, and propylene glycol, are usually added to the clinical formulation. In addition to the design of new injection formulations, the bioavailability of puerarin can be ameliorated by applying specific drug delivery systems that include microemulsions, nanoparticles, and nanocrystals [5]. Moreover, structural modifications to increase the water solubility and liposolubility are also considered. Currently, structural modifications of puerarin mainly include modifications on the phenolic hydroxyl group at position 4' and 7,4', the alcohol hydroxyl group at position 6', and a modification at the 3',5' position [6].

## 3. Bioactivity of Puerarin in Central Nervous System Disorders

Since the therapeutic potential of puerarin for CNS disorders has recently attracted considerable attention, it is essential to understand its bioactivity and determine its pharmacological action.

### 3.1 Alzheimer’s Disease

AD is the most common neurodegenerative disease and is a major form of dementia. AD is characterized by progressive cognitive impairment and memory loss [7], however, the etiology and pathogenesis of AD are not yet fully understood. Typical pathological features of AD consist of extracellular senile plaques resulting from dysregulation of amyloid-beta (Aβ) metabolism and intracellular neurofibrillary tangles formed by hyperphosphorylated microtubule-associated protein tau [8,9]. In addition, synaptic and neuronal loss [10], neuroinflammation [11], altered brain glucose metabolism [12], mitochondrial dysfunction [13], oxidative stress [10], and dysregulated neural circuits [14,15] have also been observed in human patients and animal models of AD. So far, there has been no effective intervention to block or reverse AD progression [16]. Although sodium oligomannate (GV-971), shown previously to restore gut microbiota and alleviate neuroinflammation [17], has recently been approved in China for the treatment of mild to moderate AD [18], further experimental and clinical evidence is needed to confirm its pharmacological activity.

Accumulating evidence suggests that puerarin exerts substantial neuroprotective effects through various mechanisms in AD models. Anukulthanakorn et al. demonstrated that treatment with 7 mg/kg puerarin for 120 days ameliorated cognitive impairment in ovariectomized rats. The mechanism of action could be partly associated with the inhibition of amyloid precursor protein (APP), β-Secretase 1 (BACE1) and tau4, which are associated with the formation of amyloid plaques and tau hyperphosphorylation [19]. Similar results were also observed in a *Drosophila melanogaster* AD model, where Ahuja et al. demonstrated that puerarin may serve as a potential BACE1 inhibitor to rescue cognitive decline [20]. Moreover, it was also reported that both *Puerariae radix* aqueous extract and puerarin alleviated cognitive impairment in an Aβ_25-35_-induced AD model by decreasing the levels of Aβ deposition and hyperphosphorylated tau protein, as well as by preventing neuroinflammation and the loss of noradrenergic and serotonergic neurons [21]. In addition, puerarin markedly rescued cognitive deficits and reduced phosphorylation levels at the Thr231, Ser396, and Ser 195/198/199/202 sites of tau via the inhibition of glycogen synthase kinase-3β (GSK-3β) in several AD animal models [22-24]. These findings imply that puerarin could alleviate cognitive dysfunction in AD animal models by reducing the Aβ burden and the level of tau hyperphosphorylation.

Since synaptic injury strongly correlates with cognitive dysfunction in AD, maintaining synaptic homeostasis is a promising approach for AD treatment [25]. A recent study reported that puerarin increased synaptic thickness, density, and length, and relieved the calcium overload in the hippocampus and cortical neurons in Aβ_25-35_-induced AD rats. This effect could possibly be attributed to the increased levels of calcium/calmodulin-dependent protein kinase IIα (CaMKIIα) and the activation of the p38 mitogen activated protein kinase-cyclic-adenosine monophosphate (cAMP) response element-binding protein (MAPK-CREB) signaling pathway, which is essential for synaptic plasticity and memory formation [26]. In addition, puerarin was able to enhance the extension of axons and dendrite lengths, the numbers of neuronal arbors, and synapse formation due to upregulation of a number of proteins involved in neurite or synaptic development, including dynein light chain 2, elongation factor 2, and actin-related protein 2 [27,28].

Of note, altered iron metabolism also plays a significant role in the pathogenesis of AD [29]. Since increased iron content was observed in the brains of AD animals and patients, achieving iron homeostasis may provide a promising perspective in the development of novel medications against AD [30]. In fact, Yu et al. demonstrated that puerarin ameliorated cognitive and memory deficits in APP/PS1 mice by affecting the changes of the expression level of iron metabolism-related proteins. On the one hand, puerarin downregulated the expression of iron uptake proteins, including divalent metal transporter 1 with or without iron response element, transferrin, transferring receptor 1, iron storage protein, ferrtin, and iron regulated hormone, hepcidin. On the other hand, it upregulated the expression of iron release protein, including ferroportin 1, ceruloplasmin, and hephaestin [31]. Furthermore, puerarin could alleviate iron overload in the cerebral cortex in APP/PS1 transgenic mice by reducing inflammation and oxidative stress as evidenced by the reduced level of interleukin 1β, interleukin 6, and tumor necrosis factor α (TNF-α), as well as that of glutathione peroxidase (GSH-Px), superoxide dismutase (SOD), and malondialdehyde (MDA) [32]. Additionally, puerarin inhibited Aβ_1-40_-induced NOD-like receptor family, pyrin domain containing 3(NLRP3) inflammasome activation, which was triggered by reactive oxygen species (ROS)-dependent oxidative stress through the activation of the nuclear factor E2-related factor 2 (Nrf2)/heme oxygenase-1 (HO-1) antioxidant signaling pathway [33].

Apoptosis, a biological process referring to preprogrammed cell death, plays a vital role in tissue homeostasis, the elimination of damaged cells, and aging [34]. In particular, hyperactive neuronal apoptosis, being a result of various cellular events, such as Aβ deposition, tau hyperphosphorylation, Bcl2, Bax, and caspases activation, neuroinflammation, and oxidative stress, can also lead to deleterious neurodegenerative disorders such as AD or PD [35]. Mounting evidence supports the fact that puerarin can attenuate cognitive impairment in AD mouse models through the suppression of apoptosis via activation of the phosphatidylinositol 3-kinase (PI3K)/Aktsignaling pathway [36-38], down-regulation of the Bax/Bcl-2 ratio, inhibition of c-Jun N-terminal Kinase (JNK), p38, and caspase-3 [39,40], and activation of estrogen receptor β [41]. A recent proteomics study revealed that inhibition of extracellular signal-regulated kinases 1 and 2 (ERK1/2), cyclase-associated protein 1, and Bax also mediated the anti-apoptotic activity of puerarin [42]. Besides, oxidative stress has also been recognized as a contributing factor in the progression of AD. Increased production of ROS can directly impair synaptic plasticity, thus leading to cognitive dysfunction [43]. Several studies reported that puerarin treatment reversed cognitive deficits in AD models by preventing excessive ROS production and neuronal death via the inhibition of the GSK-3β/Nrf2 and inducible nitric oxide synthase (iNOS)/ nitric oxide (NO) pathways [44,45], as well as activation of the PI3K/Akt/eNOS pathway [46].

**Figure 2.**
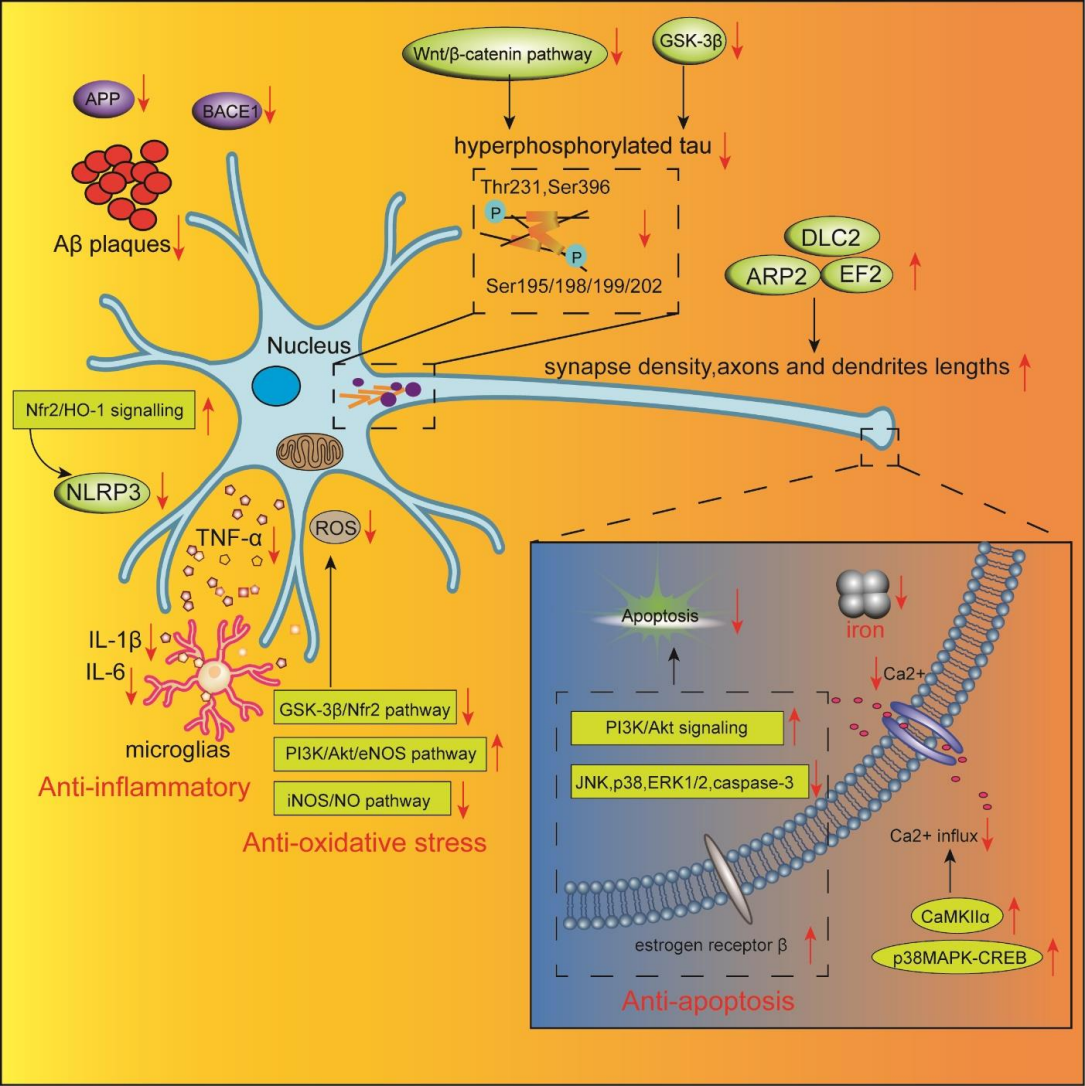
**Schematic representation of neuroprotective effects of puerarin in AD**. Red downward arrow represents inhibitory effects, while red upward arrow represents stimulative effects.

Taken together, these findings support the hypothesis that puerarin exerts its neuroprotective effects in AD through multiple pathways (Fig. 2) and can potentially be a novel drug candidate for AD treatment. However, the existing studies are mainly limited to observational research regarding the effects of puerarin on well-known AD pathologies, such as β-amyloid plaques formation, tau hyperphosphorylation, apoptosis, oxidative stress or glia activation, and are much less focused on the investigation of its underlying mechanisms. Thus, more advanced approaches that include transcriptomics, proteomics, and metabonomics should be applied to validate the biological and pharmacological activity of puerarin in AD. Finally, since puerarin also exhibits a positive effect on glucose metabolism and gastrointestinal functions [1], it should be further investigated whether it could rescue cognitive deficits in AD by ameliorating a dysregulated cerebral glucose metabolism or remodeling altered gut microbiota.

### 3.2 Parkinson's Disease

PD is the second most common neurodegenerative disease affecting approximately 0.3% of the overall population worldwide [47]. The defining neuropathological features of PD are intracellular misfolding and aggregation of α-synuclein and neuronal loss in the substantia nigra (SN), which results in striatal dopamine deficiency [48]. PD manifests with the presence of bradykinesia together with either rest tremor or rigidity, and other non-motor symptoms such as rapid eye movement sleep behavior, depression, cognitive impairment, anosmia, or constipation [49]. The underlying pathogenesis of PD involves multiple mechanisms, including the disruption of α-synuclein proteostasis, neuroinflammation, altered brain glucose metabolism, mitochondrial dysfunction, oxidative stress, dysfunction of calcium homeostasis and axonal transport, which are in part similar to those of AD. Treatment of PD includes pharmacological substitution of deficient striatal dopamine and non-dopaminergic approaches for both motor and non-motor symptoms, however, a potential disease-modifying therapy still remains a challenge [50].

There is accumulating evidence indicating that puerarin could be a promising candidate for PD treatment due to its neuroprotective properties. Results from Zhao et al. demonstrated that puerarin effectively ameliorated motor abnormalities in 1-methyl-4-phenyl-1,2,3,6-tetrahydropyridine (MPTP)-lesioned mice (commonly used PD neurotoxin model), promoted neurite outgrowth, and enhanced the survival of dopaminergic neurons against MPTP neurotoxicity by increasing progesterone receptor signaling-mediated transcriptional activity [51]. In addition, puerarin attenuated dopaminergic neuronal degeneration in the lesioned SN that was induced by 6-hydroxydopamine (6-OHDA) through the regulation of endogenous brain-derived neurotrophic factor (BDNF) expression [52]. Aside from this, Zhao et al. found that puerarin could potentiate nerve growth factor-mediated neuritogenesis by more than 10-fold by activating the ERK1/2, PI3K/Akt, and Nrf2/ HO-1 pathways [53].

**Figure 3.**
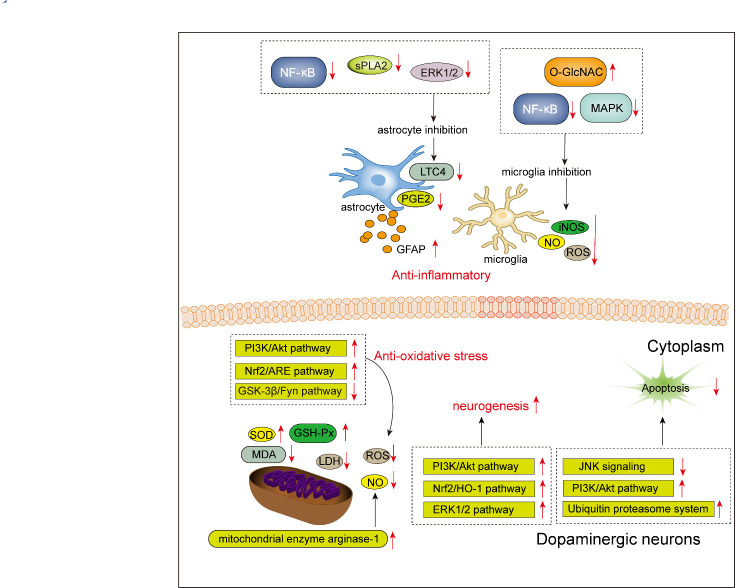
**Schematic representation of neuroprotective effects of puerarin in PD**. Red downward arrow represents inhibitory effects, while red upward arrow represents stimulative effects.

Growing evidence suggests that neuroinflammation is an important contributor to the progressive degeneration of dopaminergic neurons in PD [54]. Jiang et al. reported that after puerarin treatment the expression level of glial fibrillary acidic protein (GFAP, a marker for astrocyte activation) and iNOS was decreased, and that dopaminergic neuron loss in the SN was rescued in an MPTP-induced PD mouse model, indicating that puerarin may be a promising dopaminergic neuroprotective drug [55]. Moreover, it was shown that acetylpuerarin inhibited astrocyte activation by downregulating group V secretory phospholipase A2 (sPLA2), cytosolic PLA2 alpha (cPLA2α), nuclear factor-κB (NF-κB), and phosphorylation of ERK1/2, thus preventing the production of prostaglandin E2 and leukotriene C4 in astrocytes [56]. Further studies demonstrated that puerarin effectively suppressed microglia activation by inhibiting the expression of iNOS, and the production of NO and ROS, which were mediated by O-linked-N-acetylglucosaminylation (O-GlcNAcylation), MAPK phosphorylation, and NF-κB translocation [57].

In addition to anti-neuroinflammatory potential, puerarin also displays anti-oxidative activity. It is commonly accepted that mitochondrial dysfunction and oxidative stress contribute to the molecular pathogenesis of PD [58]. Since mitochondria are the main sites of ROS production and are particularly susceptible to oxidative stress-induced damage [59], the disrupted balance between the production and elimination of ROS results in cellular dysfunction and, ultimately, in the death of dopaminergic neuron in PD. Several studies have reported that a protective role of puerarin against oxidative stress injury exists. In particular, this role is likely due to increasing SOD and glutathione (GSH) activities, decreasing MDA activity, reducing ROS and LDH generation [60], preserving mitochondrial membrane potential, and preventing cytochrome c release [61]. Zhu et al. proved that puerarin alleviated MPTP-induced dopaminergic neuron degeneration and depletion by upregulating glial cell line-derived neurotrophic factor (GDNF) expression, and activating the PI3K/Akt pathway and GSH, which subsequently reduced MPTP-induced ROS production [62]. Besides, puerarin could suppress nuclear exclusion of Nrf2 by inhibiting the GSK-3β/Fyn pathway, which in turn, induced antioxidant response element (ARE)-driven glutamate cysteine ligase gene transcription and increased its *in vitro* and *in vivo* synthesis, thus attenuating MPP+/MPTP-induced oxidative stress [63]. Similarly, activation of the Nrf2/ARE signaling pathway was also shown to be involved in the anti-oxidative effect of puerarin [52]. Moreover, Zhao et al. demonstrated that puerarin attenuated 6-OHDA-induced NO production and neurotoxicity by increasing mitochondrial enzyme arginase-2 expression in midbrain neurons [64].

Additionally, puerarin was found to prevent dopaminergic neuron loss through the inhibition of apoptosis. This anti-apoptotic effect could be attributed to the inhibition of the JNK signaling pathway [65], activation of the PI3K/Akt pathway [66], and upregulation of G protein-coupled receptor 30 and GDNF [67]. Cheng et al. found that puerarin could protect MPP^+^-induced SH-SY5Y cells from apoptosis by attenuating the dysfunction of ubiquitin proteasome system [68].

In summary, experimental evidence indicates that puerarin could prevent dopaminergic neuron degeneration by exerting anti-inflammatory, anti-oxidative, anti-apoptotic, and pro-neurogenic effects (Fig. 3). As was the case with AD, certain challenges should be addressed to validate the bioactivity of puerarin in PD.

### 3.3 Cerebral Ischemia

Cerebral ischemia triggers cellular bioenergetic failure as a result of focal cerebral hypoperfusion, followed by blood-brain barrier (BBB) dysfunction [69], oxidative stress injury [70], neurovascular unit injury [71], excitotoxicity [72], post-ischemic neuroinflammation, and finally the death of neurons and glia [73,74]. Experimental evidence suggests that puerarin can protect the brain from cerebral ischemia injury through multiple mechanisms. For instance, Kong et al. investigated the distribution kinetics of puerarin in the rat hippocampus after cerebral ischemia and found that the area under the curve (AUC_0-120min_) and the maximum concentration (C_max_) of puerarin in the embolic hippocampus were higher than those of the normal hippocampus [75], indicating that puerarin accumulation was selective towards ischemic areas. In addition, delayed puerarin treatment (starting 24 hours after focal ischemic stroke) demonstrated long-term therapeutic effects, which could be partially explained by enhanced vascular remodeling [76]. Besides, the elimination rate of puerarin in a cerebral ischemia reperfusion rat model was slower than that in a healthy rat [77].

It is well-known that cerebral ischemia mainly induces bioenergetic failure [73], oxidative stress [70], calcium overload and neuronal apoptosis [78]. It was found that puerarin could improve cerebral blood perfusion by p42/44 MAPKs-mediated angiogenesis [79]. Lyophilized powder of puerarin and catalpol (the bioactive component isolated from *Rehmannia glutinosa*) not only increased regional cerebral blood flow, reduced infarct volume and protected vessel integrity in cerebral artery occlusion rats, but also inhibited brain vascular endothelial cell apoptosis by upregulating hypoxia-inhibitory factor-1α(HIF-1α) that was dependent on the ERK and PI3K/Akt/mammalian target of rapamycin (mTOR) signaling pathways [80]. Furthermore, acidosis is a common feature in cerebral ischemia, which can aggravate ischemic brain injury [81]. It has been demonstrated that puerarin protected the rat brain against acidosis-induced injury after cerebral ischemia by inhibiting acid sensing ion channel 1a, which was activated by extracellular acidosis, and could facilitate the activation of voltage-gated Ca^2+^channels and intracellular Ca^2+^accumulation [82]. Additionally, cognitive impairment and anxiety-like behavior induced by cerebral ischemia could be alleviated by puerarin due to the activation of the PI3K/Akt1/GSK-3β/ myeloid cell leukemia-1 (MCL-1) signaling pathway, and the reduction of MDA, GSH-Px and thiol levels in the hippocampus and frontal cortex [83]. Nrf2, Forkhead boxO1(FoxO1), Forkhead box O3(FoxO3), and Forkhead box O1(FoxO4) involved in antioxidant effects were also upregulated, thus decreasing ROS production [84]. Zhao, et al. confirmed the antioxidant effect of puerarin in several cerebral ischemia-reperfusion injury (CI-CR) animal models, which was supported by reduced levels of lactic acid (LA), lipid peroxide, and increased levels of GSH-Px and Na^+^-K^+^-ATPase [85]. Moreover, calcium overload, leading to continuous NO production, has been reported to trigger neuronal death following cerebral ischemia [86]. Zhang et al. showed that puerarin repressed calcium overload in a rat model of transient focal ischemia [87]. Another study indicated that puerarin decreased the level of excitatory neurotransmitter glutamate and NO, downregulated oxygen-glucose deprivation-induced Ca^2+^ influx and the intracellular Ca^2+^ peak, thus inhibiting the apoptotic cascade [88]. Multiple studies also demonstrated the role of puerarin against cerebral ischemia injury by attenuating autophagy through the activation of the APMK-mTOR-Unc-51-like kinase 1 (ULK1) signaling pathway [89,90]. It has also been shown that puerarin reduced cerebral edema in CI-CR in part through the suppression of HIF-1α and activation of TNF-α, followed by the inhibition of iNOS and caspase-3 [91].

**Figure 4.**
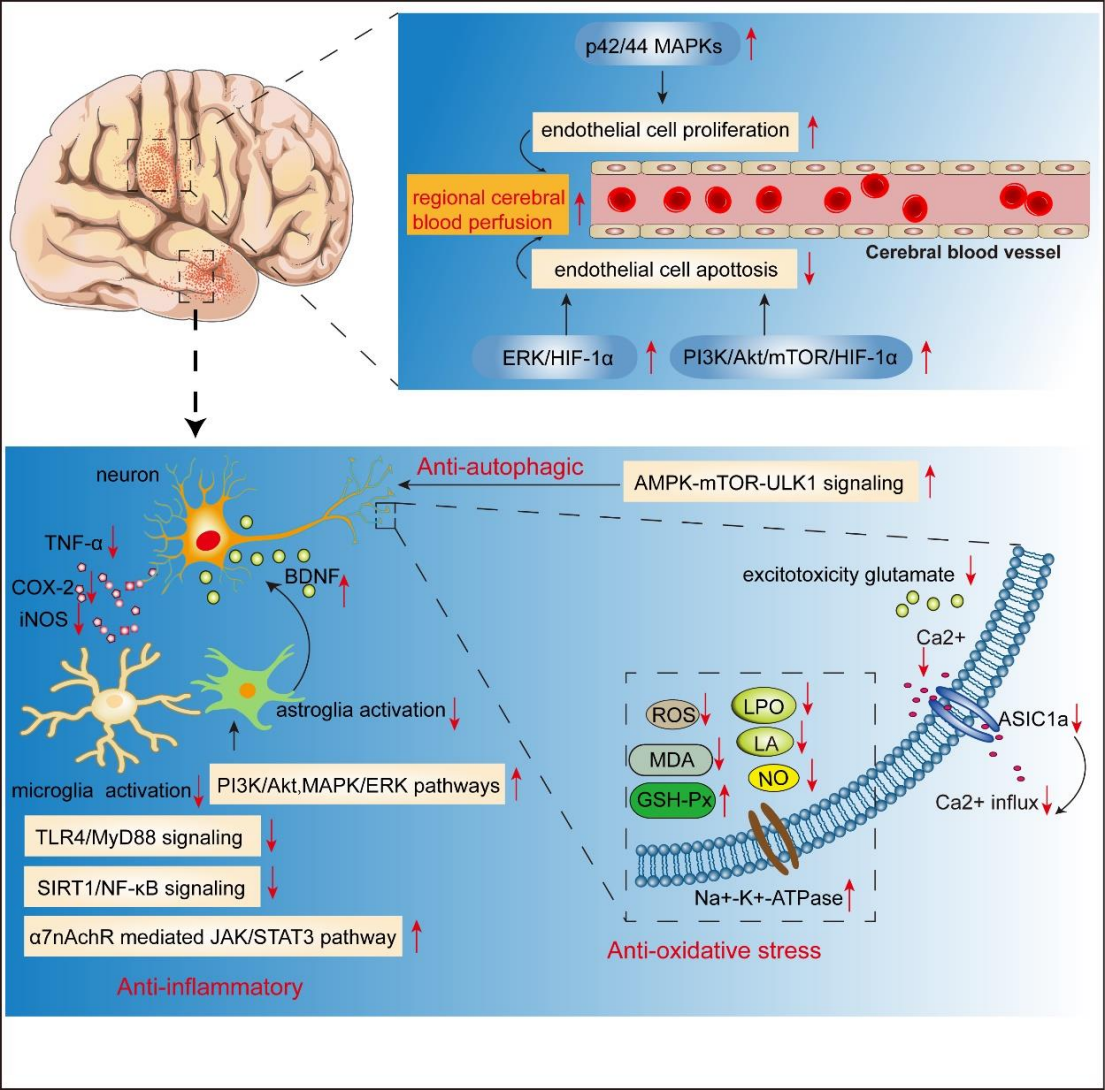
**Schematic representation of neuroprotective effects of puerarin in cerebral ischemia**. Red downward arrow represents inhibitory effects, while red upward arrow represents stimulative effects.

Moreover, the anti-inflammatory effect of puerarin in CI-RI has been investigated. Puerarin (at a dose of 100 mg/kg) reduced the brain infarct volume and improved neurological deficits by suppressing astrocyte and microglia activation, as well as multiple inflammatory factors such as cyclooxygenase-2 [92], TNF-α andToll-like receptor 4 (TLR4)/Myeloid differentiation primary response 88 (MyD88), and the silent information regulator 1 (SIRT1) /NF-κB pathway [93,94]. Importantly, astrocytes provide structural, trophic, and metabolic support for neurons, and play a significant role in neuronal survival and plasticity after cerebral ischemia injury [95]. Wang et al. reported that puerarin protected the brain from cerebral ischemia injury by inhibiting astrocyte apoptosis and enhancing BDNF secretion by astrocytes, which was associated with activation of the PI3K/Akt and MAPK/ERK signaling pathways [96]. In addition, intravenous injection of puerarin attenuated the inflammatory response in CI-RI rats by activating the α7nAchR-mediated Janus kinase 2 (JAK2)/signal transducer and activator of transcription 3 (STAT3) cholinergic anti-inflammatory pathway [97].

In conclusion, these findings indicate that puerarin may protect the brain from cerebral ischemia injury by enhancing neurogenesis, increasing cerebral blood perfusion, and by exerting anti-apoptotic, anti-inflammatory, anti-oxidative, and anti-autophagic properties, as well as alleviating excitotoxicity (Fig. 4).

**Figure 5.**
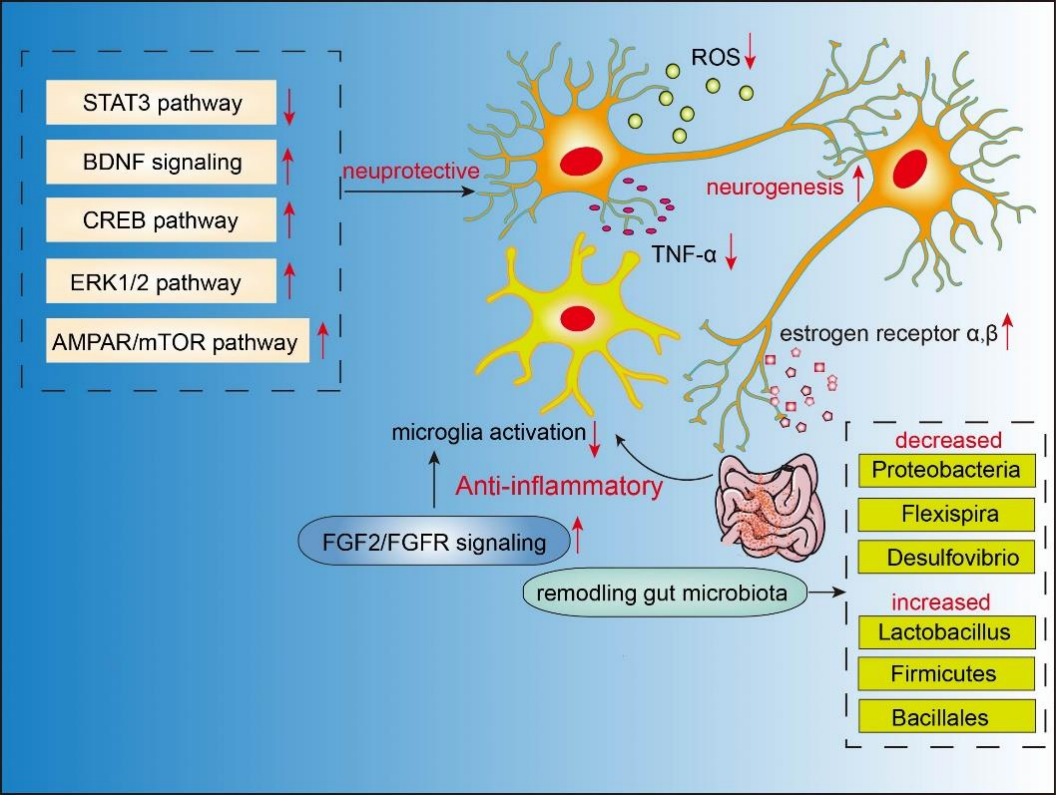
**Schematic representation of neuroprotective effects of puerarin in depression**. Red downward arrow represents inhibitory effects, while red upward arrow represents stimulative effects.

### 3.4 Depression

Depression is a common chronic mental disorder that affects a growing population across the globe. Clinically, depression is characterized by persistent and recurrent low self-esteem, low mood, diminished interests, impairments in cognition, and even suicidal ideation. Current medication-based therapies consist of antidepressants that include selective serotonin reuptake inhibitors, tricyclics tetracyclics, and monoamine oxidase inhibitors. However, their long-term use may cause a wide range of adverse events such as autonomic dysfunction, serotonin syndrome, liver toxicity, and cardiovascular diseases [98].

In recent years, puerarin has gained increasing attention for its antidepressant properties, and can thus be regarded as a promising candidate for the treatment of depression. Various studies have demonstrated that puerarin exerts anti-depressive effect by inhibiting apoptosis, decreasing ROS production, increasing expression levels of AKT1 and FOS, and reducing expression of caspase-3, STAT3, and TNF-α, which correlated with depression signaling pathways [99]. In addition, puerarin ameliorated spared nerve injury-induced depression and pain in mice by activating the ERK1/2, CREB, and BDNF pathways [100]. A recent study demonstrated that puerarin could promote neurogenesis and attenuate microglia activation by triggering fibroblast growth factor-2 (FGF-2)/ fibroblast growth factor receptor (FGFR) signaling [101]. Similarly, it has also been shown that puerarin ameliorated ovariectomy-induced depressive-like behavior possibly through enhancing neurogenesis in the dentate gyrus of the hippocampus, and upregulating BDNF and the transcription of various estrogen receptors (ERα and ERβ) [102]. Similarly, activation ofα-amino-3-hydroxy-5-methylisoxazole-4-propionic acid receptors (AMPAR)/ mTOR signaling, together with an increased release of BDNF [103], upregulation of BDNF and activation of ERK signaling in the prefrontal cortex in diabetic rats [104], contributed to the anti-depressive effects of puerarin.

It is commonly accepted that altered gut microbiota homeostasis correlates with depression [105,106]. A very recent study investigated the changes in the gut microbiota composition in chronic unpredictable mild stress mice upon puerarin treatment [107]. Puerarin alleviated depression-like behavior in mice, and importantly, decreased the abundance of pro-inflammatory bacteria such as Proteobacteria, Flexispira, and Desulfovibrio. Interestingly, the abundance of anti-inflammatory bacteria such as Firmicutes, Bacillales, and Lactobacillus was increased, indicating that puerarin could ameliorate depressive behavior by remodeling dysregulated gut microbiota [107]. Putative puerarin mechanisms of action in depression are illustrated in theFig. 5.

### 3.5 Spinal Cord Injury

In addition to the potential therapeutic effects in AD, PD, and CI-RI, puerarin has been found to promote spinal cord injury repair. It was demonstrated that the optimal timing for puerarin treatment in spinal ischemic damage was within 4 hours of spinal ischemia-reperfusion injury, which resulted in an increase in thioredoxin transcription and inhibition of the apoptosis [108]. Glutamate dysregulation plays a central role during spinal ischemic injury and subsequent reperfusion, triggering damage and death of nerve cells [109]. Tian et al. reported that puerarin reduced acute spinal cord injury by predominantly inhibiting metabotropic glutamate receptor transcription and glutamate release [110]. In line with this, puerarin showed neuroprotective effects in rats against acute spinal cord injury through the suppression of glial activation and apoptosis, which might be associated with activating the PI3K/Akt signaling pathway [111]. Moreover, the inhibition of cyclin-dependent kinase 5(Cdk5) and p25, which play a prominent role in apoptosis, also contributed to the neuroprotective activity of puerarin in acute ischemia/reperfusion-induced spinal injury [112]. In addition, anti-oxidative and anti-apoptotic properties of puerarin exhibited protective effects on secondary spinal cord injury, as was evidenced by an increase in SOD activity and the Bcl/Bax ratio, as well as decreased MDA expression [113]. Furthermore, suppressing oxidative stress by inhibiting the p38 MAPK pathway was shown to occur in response to puerarin and resveratrol-loaded nanoparticles [114].

## 4. Conclusion and Future Perspectives

As summarized and discussed in this review article, puerarin demonstrated neuroprotective effects through multiple pathways in various CNS disorders, including AD, PD, cerebral ischemia, depression, and spinal cord injury. The underlying mechanisms of action of puerarin are associated with anti-apoptotic, anti-oxidative, anti-autophagic, anti-inflammatory, and pro-neurogenic mechanisms. This scientific evidence indicates that puerarin could be a promising candidate compound for the treatment of various CNS diseases. Nevertheless, previous research aimed at investigating the neuroprotective mechanisms of puerarin mainly focused on a single signaling pathway without considering broader associations with other biological processes, leading to potentially incomplete evidence. To broaden the understanding of the potential mechanisms associated with puerarin neuroprotection, bioinformatics analyses and multi-omics technologies, including genomics, transcriptomics, proteomics, and metabolomics should be employed. Besides, the clinical use of puerarin is limited due to its low solubility in water and lipids, which may impair its permeability through the BBB and its pharmacological activity. Therefore, identification of puerarin derivatives with improved penetration and bioavailability should be considered. For instance, Ji et al. reported that puerarin derivatives with improved log P values were more lipophilic, and hence passed more efficiently through the BBB, which led to a stronger inhibition of the inflammatory responses and enhanced Ca^2+^-Mg^2+^-ATPase activity in CI-RI [115]. Additionally, drug delivery and transportation systems technology, such as nanoparticles and liposomes, hold great potential to facilitate new formulations. Specifically, puerarin-loaded hydroxypropyl beta cyclodextrin nanoparticles not only increased and prolonged puerarin concentration in the brain, but also markedly decreased the infarction volume after the administration of puerarin-derived nanoparticles in CI-RI rat brain [116]. Moreover, puerarin-loaded poly(butylcyanoacrylate) nanoparticles coated with polysorbate 80 fabricated by Zhao et al. showed higher concentrations and exhibited stronger neuroprotective effects against CI-RI than free puerarin [117]. These findings indicate that drug delivery and transportation systems possess great potential for the clinical application of puerarin. Of note, the optimization of drug carrier materials for better penetration through the BBB and bioavailability of puerarin requires collaborative multidisciplinary approaches between neuroscience, toxicology, pharmacology, and material science.

Results from a recent clinical trial showed that the combined treatment of puerarin and naloxone exhibited better efficacy in patients with traumatic cerebral infarction than a conventional therapy [118]. Another trial also reported that dual therapy with puerarin and aspirin improved neurological functions in patients with acute cerebral infarction, along with decreased levels of von Willebrand factor and thrombomodulin, indicating damaged vascular endothelial cells present in the blood serum [119]. However, the evidence supporting the therapeutic efficacy of puerarin on survival or dependency in people with ischaemic stroke is still inconclusive [120,121]. Therefore, well-designed and large-scale randomised controlled trials with long-term follow-ups are required to validate the efficacy of puerarin in cerebral ischemia. Since puerarin-based mechanisms of action are not fully elucidated and because of the lack of standard dosing, its clinical efficacy in other CNS diseases has not yet been validated. In addition, only few studies have so far been performed to evaluate the toxicity of puerarin [122], and in the future it will be crucial to examine in more detail its potential hepatic and renal toxicity. Thus, future investigation should focus on the exploration of the pharmacological mechanisms of action of puerarin, its toxicity and high-quality clinical research. The extensive neuroprotective properties of puerarin, the bioactive ingredient isolated from *Pueraria lobata*, provide new insights and perspectives for the discovery and development of novel medications for the management of CNS disorders.
